# *TCF21* is related to testis growth and development in broiler chickens

**DOI:** 10.1186/s12711-017-0299-0

**Published:** 2017-02-24

**Authors:** Hui Zhang, Wei Na, Hong-Li Zhang, Ning Wang, Zhi-Qiang Du, Shou-Zhi Wang, Zhi-Peng Wang, Zhiwu Zhang, Hui Li

**Affiliations:** 1 Key Laboratory of Chicken Genetics and Breeding, Ministry of Agriculture; Key Laboratory of Animal Genetics, Breeding and Reproduction, Education Department of Heilongjiang Province; College of Animal Science and Technology, Northeast Agricultural University, Harbin, 150030 People’s Republic of China; 2 Department of Crop and Soil Sciences, Washington State University, Pullman, WA 99164 USA

## Abstract

**Background:**

Large amounts of fat deposition often lead to loss of reproductive efficiency in humans and animals. We used broiler chickens as a model species to conduct a two-directional selection for and against abdominal fat over 19 generations, which resulted in a lean and a fat line. Direct selection for abdominal fat content also indirectly resulted in significant differences (*P* < 0.05) in testis weight (TeW) and in TeW as a percentage of total body weight (TeP) between the lean and fat lines.

**Results:**

A total of 475 individuals from the generation 11 (G_11_) were genotyped. Genome-wide association studies revealed two regions on chicken chromosomes 3 and 10 that were associated with TeW and TeP. Forty G_16_ individuals (20 from each line), were further profiled by focusing on these two chromosomal regions, to identify candidate genes with functions that may be potentially related to testis growth and development. Of the nine candidate genes identified with database mining, a significant association was confirmed for one gene, *TCF21*, based on mRNA expression analysis. Gene expression analysis of the *TCF21* gene was conducted again across 30 G_19_ individuals (15 individuals from each line) and the results confirmed the findings on the G_16_ animals.

**Conclusions:**

This study revealed that the *TCF21* gene is related to testis growth and development in male broilers. This finding will be useful to guide future studies to understand the genetic mechanisms that underlie reproductive efficiency.

**Electronic supplementary material:**

The online version of this article (doi:10.1186/s12711-017-0299-0) contains supplementary material, which is available to authorized users.

## Background

Body weight has been intensively selected for more than half a century and will continue to be one of the most important economic traits in broiler chicken breeding programs. However, the results of previous studies showed that selection for increased growth rate often results in a number of undesirable traits, including ascites, lameness, reduced fertility, and reduced resistance to infectious diseases [1]. Selection for rapid growth has also been accompanied by increased fat deposition in chickens [2, 3]. Therefore, multiple selection criteria must be applied beyond body weight, including abdominal fat and reproduction traits.

Studies on related traits in other species, including those on obesity and fertility in humans, provide some insight towards the overall improvement of broiler production. For example, the relationship between human obesity and fertility traits has been investigated for many years and the results indicate that obesity in men is positively associated with infertility [4]. A recent study reported that androgen and sex hormone-binding globulin (SHBG) serum levels were reduced in obese men and estrogen levels were increased, without a compensatory increase in the follicle-stimulating hormone (FSH) [4].

The chicken (*Gallus gallus*) is an important animal model that can be used to bridge the evolutionary gap between mammals and other vertebrates [5]. Two broiler lines at the Northeast Agricultural University (China) were divergently selected for 19 generations for abdominal fat content (named the NEAUHLF lines), one for high abdominal fat (fat line) and one for low abdominal fat (lean line) [see Additional file 1: Table S1]. Starting from generation 4, the two lines displayed significant differences in abdominal fat in 7-week-old birds [6]. Excessive fat deposition not only decreases feed efficiency but is also associated with decreases in reproductive efficiency in male and female chickens [7, 8]. A previous study on chickens indicated that small testes were often associated with poor fertility [9].

In this study, we focused on testis weight (TeW) and testis percentage of body weight at 7 weeks of age (TeP = TeW/BW7) in broiler chickens to: (1) conduct genome-wide association studies (GWAS) to identify regions that harbor genes controlling testis growth, (2) identify candidate genes in these regions, and (3) profile candidate genes for future cloning studies. We were able to identify genetic loci that are involved in the reproduction of broilers, which could be useful in targeted breeding strategies to accelerate the breeding of healthy, high-quality broilers.

## Methods

### Ethics statement

All experiments involving animals were conducted according to the guidelines for the care and use of experimental animals, established by the Ministry of Science and Technology of the People’s Republic of China (Approval Number: 2006-398). Experimental animal work was also approved by the Laboratory Animal Management Committee of the Northeast Agricultural University.

### Genome-wide association study (GWAS)

Broiler chickens used in this study were from two Northeast Agricultural University broiler lines, divergently selected for abdominal fat content (named NEAUHLF lines). NEAUHLF lines have been selected since 1996 using abdominal fat percentage (AFP) [AFP = abdominal fat weight (AFW)/body weight at 7 weeks of age (BW7)] and plasma very low-density lipoprotein (VLDL) levels as selection criteria. Details on these two lines are in Zhang et al. [10]. The body weight of the male birds at 1, 3, 5, and 7 weeks of age (BW1, 3, 5, and 7) were measured. The male birds were slaughtered at 7 weeks of age and testis weight (TeW), testis percentage of BW7 (TeP = TeW/BW7), AFW (abdominal fat weight), and AFP (abdominal fat percentage) were recorded. A total of 475 individuals from generation 11 (G_11_), which was the latest generation at that time, were used in the genome-wide association studies (GWAS). Descriptive statistics of AFW, AFP, TeW and TeP were obtained by using JMP 4.0 [11].

Genomic DNA was extracted from blood by using the classical phenol/chloroform method and diluted to 50 ng/µL. The quality and concentration of genomic DNA fulfilled the requirements for the Illumina Infinium SNP genotyping platform. Genotyping using the Illumina 60 k Chicken SNP BeadChip was carried out at the Illumina-certified service provider, DNA LandMarks Inc., Canada. Quality controls were assessed with GenomeStudio v2008.1 [12]. Of the 57,636 SNPs, 45,611 had a minor allele frequency (MAF) higher than 5% and a call rate of 95% in the combined dataset for birds from the lean and fat lines and were retained for further analyses. Individuals with a pedigree error of more than 5% or with missing SNP genotypes were removed. The SNPs used in the current study [see Additional file 2: Table S2] were previously described in detail by Zhang et al. [10].

GWAS was carried out in PLINK using the linear regression analysis method [13]. We have performed multiple analyses to eliminate the potential false positives due the confounding with subpopulation or contemporary groups. Three models including line (fat and lean), the first two principal components (PC), and the first three PC as covariates, respectively, were used to carry out the GWAS. BW7 was also used as a covariate for TeW. Polygenetic effects were predicted by using the mixed linear model with variance and covariance structure defined by the kinship derived from all the markers. The results of the third model which included three PC as covariates are presented in the current study. We used the genome-wide 5% type I error after Bonferroni correction as the genome-wide significance level. For two traits and 45,611 SNPs, the threshold *P* value for declaring genome-wide significance was 0.05/(2 × 45,611) = 5.48 × 10^−7^. The Manhattan plots of the *P* values for all SNPs associated with TeW and TeP were plotted using SNPEVG1 version 2.1 [14]. Gene locations and information were mined from the Ensembl chicken genome galGal 3 [15].

GWAS were also carried out with mixed-model statistical packages, including GAPIT (version 1) [16], Efficient Mixed-Model Association eXpedited (EMMAX beta) [17], the Genome-wide Rapid Association using Mixed Model and Regression (GRAMMAR) approach, which is implemented in GenABEL version 1.8-0 [18], and Genome-wide Efficient Mixed-Model Association (GEMMA version 0.94) [19], using line (fat, lean) and BW7 as covariates for TeW, and line as the covariate for TeP. The random effects refer to polygenic effects of individuals, which are known as animal genetic effects. The variance and covariance structure is defined by the kinship matrix derived from all the markers. The kinship is known as the genomic relationship matrix.

### mRNA expression analysis

Based on the GWAS results, nine genes that may play a role in TeW and TeP were selected for further mRNA expression analyses. Birds from two generations, G_16_ and G_19_, were used to carry out the mRNA expression analyses and compared to assess their credibility. Forty birds (20 from the lean and 20 from the fat line) and 30 birds (15 from the lean and 15 from the fat line) were randomly chosen from the G_16_ and G_19_ populations, respectively. These birds were slaughtered at 7 weeks of age and testis (Te) tissue was isolated, weighed, immediately frozen in liquid nitrogen, and then stored at −80 °C.

Total RNA was extracted from 50 to 100 mg bulk testis tissue using Trizol reagent (Invitrogen, Carlsbad, CA, USA) according to the manufacturer’s recommendations. RNA concentrations were measured by spectrophotometry (OD at 260 nm) and their integrity was evaluated using the OD_260_/OD_280_ ratio (>1.8). cDNA was prepared with oligo (dT)-primed (Takara, Daliang, China) reverse transcription using ImProm-II Reverse Transcriptase (Promega, Madison, WI, USA). After reverse transcription, the target cDNAs were quantified by real-time PCR using an ABI 7500 system and the SYBR Green Master Mix (Roche, Basel, Switzerland). *Glyceraldehyde*-*3*-*phosphate dehydrogenase* (*GAPDH*) and *TATA box*-*binding protein* (*TBP*) were used as internal reference genes. The primer sequences used to analyze gene expression are in Table S3 [see Additional file 3: Table S3]. The PCR cycling conditions included an initial step at 50 °C for 2 min, a denaturation step at 95 °C for 10 min and 40 cycles at 95 °C for 15 s and 60 °C for 1 min. The mRNA expression levels were analyzed using the ABI 7500 software v2.0.5. Results were expressed as $2^{{ -\Delta C_{\text{t}} }}$ with Δ*C*_t_ = *C*_*tij*_ − *C*_*trj*_, where *C*_*tij*_ and *C*_*trj*_ are the *C*_t_ for target gene *i* and reference gene *r* in sample *j* [20]. Statistical significance of differences in mRNA expression levels between groups was determined with a *t* test. The correlations between the expression level of the genes and TeW and TeP was also calculated. A *P* value <0.05 was considered significant.

## Results

### Line differences in testis weight and testis percentage

**Fig. 1:**
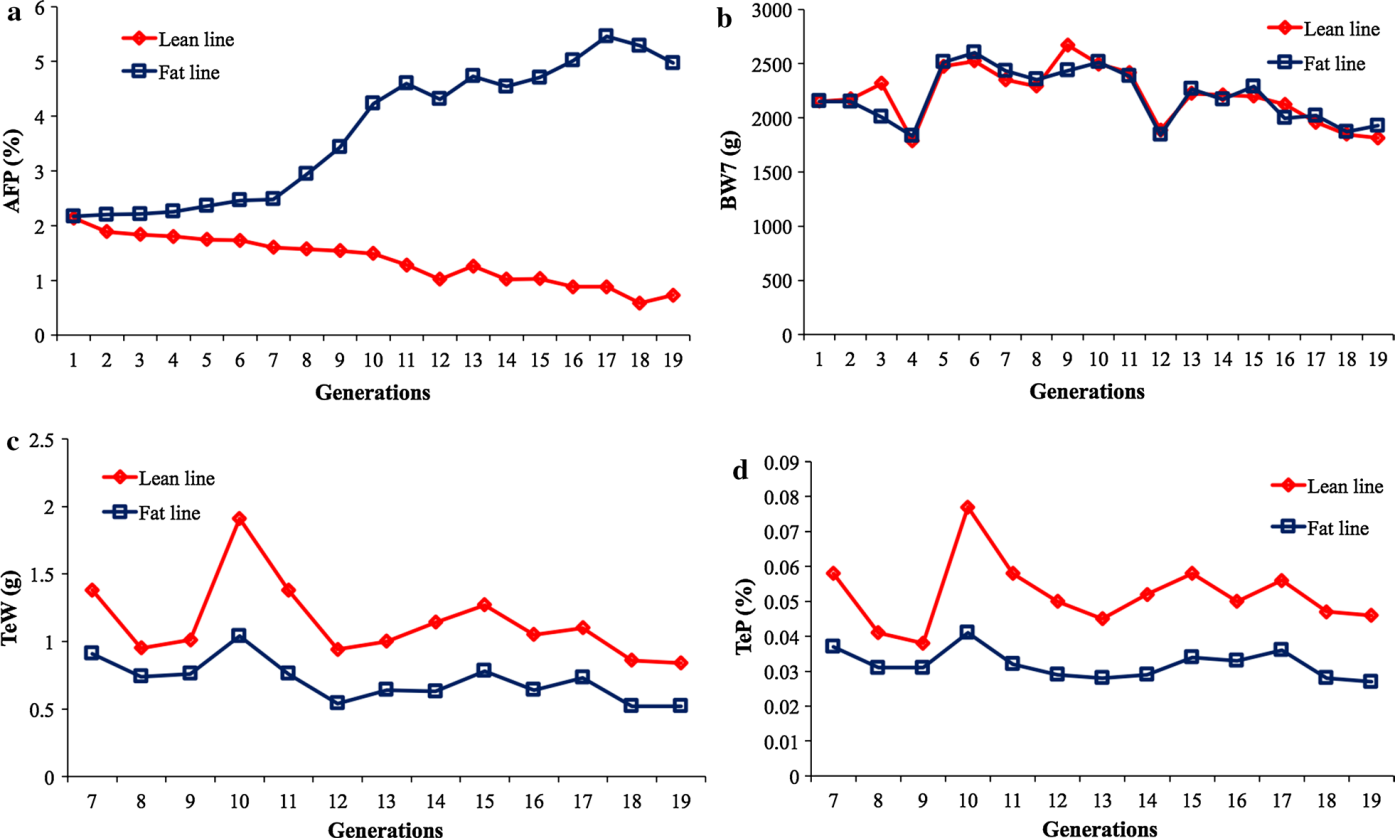
Observed phenotypic means of male broiler chickens from the *lean* and *fat lines* that were selected for abdominal fat over 19 generations. The *two lines* originated from the same population at generation 1. Starting from generation G_4_, significant differences (*P* < 0.05) in abdominal fat percentage (AFP) were observed at 7 weeks of age (**a**). A sevenfold difference was found between the *two lines* in the last generation (G_19_); however, body weight at the same age (BW7) remained similar between the *fat* and *lean lines* across all generations (**b**). Testis weight (TeW) was recorded (**c**) and testis percentage (TeP) was calculated (**d**). Both TeW and TeP exhibited significant differences between the* lean* and* fat lines* from G_7_ forward

Lean and fat broiler chicken lines were selected for 19 generations. Starting from generation 4 (G_4_), birds in the fat line had significantly higher (*P* < 0.05) abdominal fat percentages [AFP = abdominal fat weight/body weight at 7 weeks of age (BW7)] than birds in the lean line (Fig. 1a). No significant differences (*P* > 0.05) in BW7 were observed between the two lines (Fig. 1b). However, significant line differences (*P* < 0.05) were also found for TeW and TeP in G_7_ to G_19_ (Table 1; Fig. 1c, d). When these differences appeared is unclear because no phenotypic records were recorded for these two traits before G_7_. Interestingly, we also noticed that the differences between the fat and lean lines existed across generations, from G_7_ to G_19_.

**Table 1:** Least square means of TeW and TeP for the fat (*F*) and lean (*L*) lines

Generation	Individuals	TeW (L)	TeW (F)	TeP (L)	TeP (F)
7	472	1.38 ± 0.99^A^	0.91 ± 0.66^B^	0.058 ± 0.041^A^	0.037 ± 0.025^B^
8	387	0.95 ± 0.54^A^	0.74 ± 0.36^B^	0.041 ± 0.023^A^	0.031 ± 0.015^B^
9	383	1.01 ± 0.62^A^	0.76 ± 0.50^B^	0.038 ± 0.023^A^	0.031 ± 0.021^B^
10	627	1.91 ± 1.55^A^	1.04 ± 0.72^B^	0.077 ± 0.063^A^	0.041 ± 0.028^B^
11	475	1.38 ± 1.02^A^	0.76 ± 0.55^B^	0.058 ± 0.045^A^	0.032 ± 0.023^B^
12	521	0.94 ± 0.73^A^	0.54 ± 0.26^B^	0.050 ± 0.036^A^	0.029 ± 0.014^B^
13	598	1.00 ± 0.78^A^	0.64 ± 0.29^B^	0.045 ± 0.034^A^	0.028 ± 0.012^B^
14	612	1.14 ± 0.80^A^	0.63 ± 0.32^B^	0.052 ± 0.035^A^	0.029 ± 0.015^B^
15	538	1.27 ± 1.07^A^	0.78 ± 0.44^B^	0.058 ± 0.049^A^	0.034 ± 0.018^B^
16	665	1.05 ± 0.95^A^	0.64 ± 0.37^B^	0.050 ± 0.044^A^	0.033 ± 0.017^B^
17	627	1.10 ± 1.07^A^	0.73 ± 0.49^B^	0.056 ± 0.053^A^	0.036 ± 0.024^B^
18	583	0.86 ± 0.70^A^	0.52 ± 0.33^B^	0.047 ± 0.037^A^	0.028 ± 0.016^B^
19	509	0.84 ± 0.56^A^	0.52 ± 0.20^B^	0.046 ± 0.030^A^	0.027 ± 0.010^B^

Statistics include number of individuals, mean, and standard deviation. Significance tests were performed between the fat (F) and lean (L) lines for testis weight (TeW) and testis percentage (TeP), separately. For each generation, different notations (^A^ or ^B^) indicate a significant difference between the two lines at a significance level of 5%

### Genotyping

**Fig. 2:**
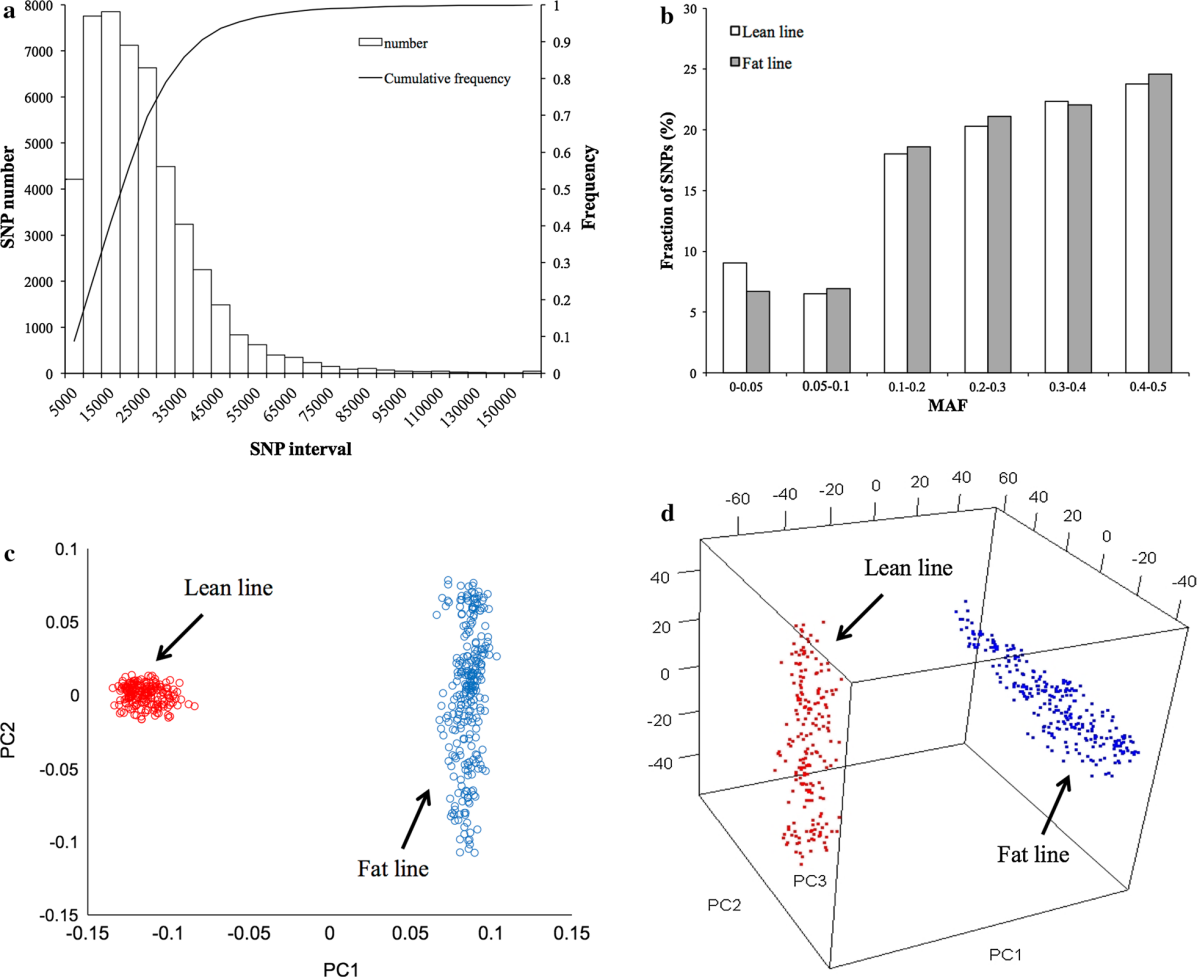
Characteristics of the genotyped SNPs and population structure. 475 individuals (203 individuals from the* lean line* and 272 from the* fat line*) were randomly selected from G_11_ for genotyping. Genotyping was performed with the Chicken-60 K Illumina chip with 80% of the intervals between adjacent SNPs <30 kb (**a**). The minor allele frequency (MAF) of the SNPs in the lean and fat lines respectively, used in the GWAS study (**b**). Principal component analysis based on these SNPs shows the separation between the *lean* and *fat lines* in a two-dimension plot (**c**) and a three-dimension plot (**d**)

The 60 k chicken SNP BeadChip, produced by Illumina Inc. at the beginning of 2009 [21], was used to conduct GWAS. A total of 475 birds from G_11_ were selected for genotyping with the 60 k chicken SNP BeadChip; 203 and 272 birds from the lean and fat lines, respectively. After quality control, 45,611 SNPs were retained for further analyses [see Additional file 2: Table S2]. The average distance between adjacent SNPs was 22 kb and 80% of the SNP intervals were smaller than 30 kb (Fig. 2a). Approximately 46% of the SNPs had a minor allele frequency (MAF) between 0.3 and 0.5 within both lines; the average MAF was 0.27 (Fig. 2b).

### Genome-wide association studies

**Fig. 3:**
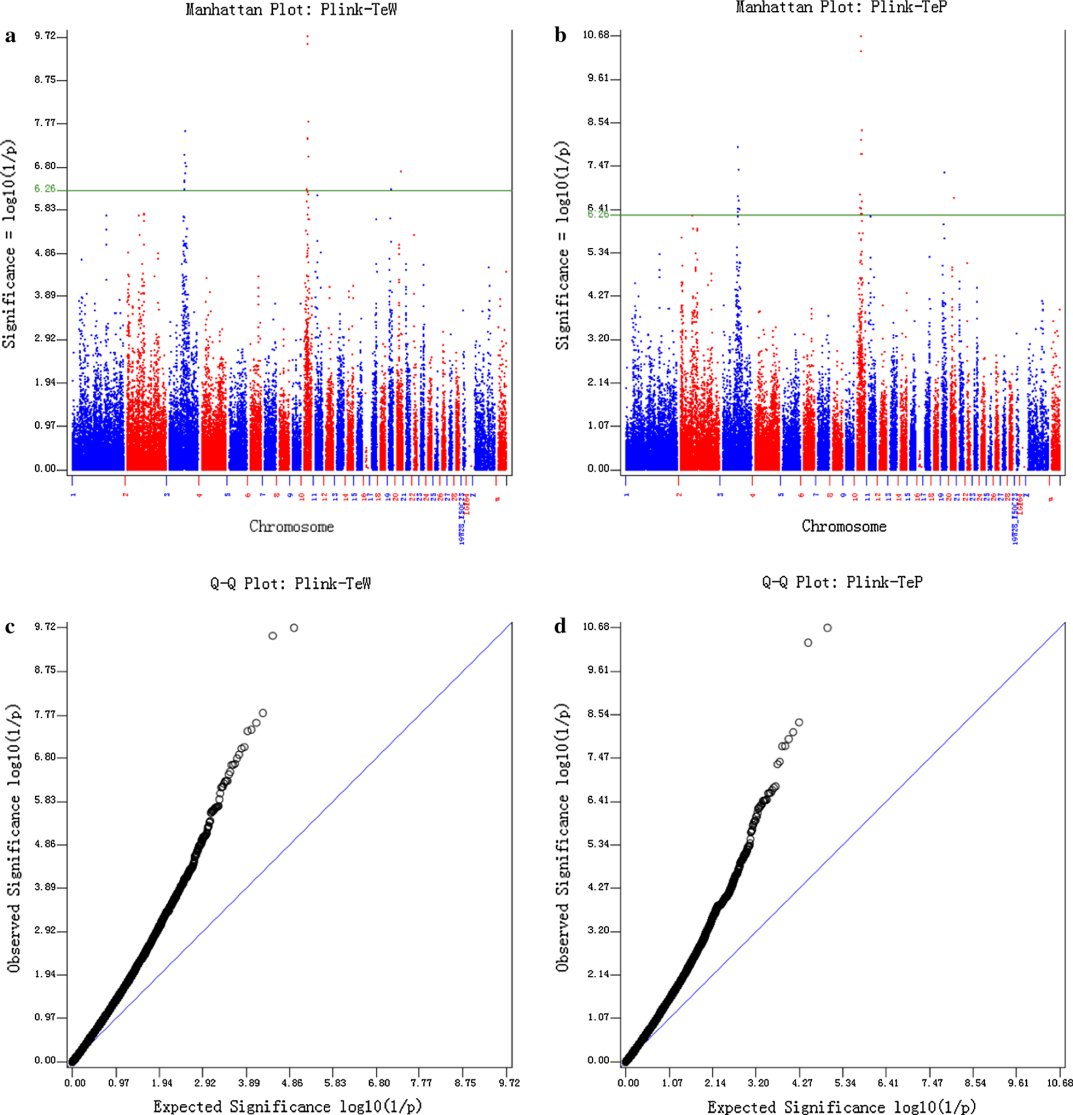
Results of genome-wide association studies using PLINK. The results are presented as Manhattan plots in the *top panels* (**a**, **b**) and *Q*–*Q* plots in the* bottom panels* (**c**, **d**). The *left panels* show the results for testis weight (TeW) and the *right panels* for testis percentage of body weight (TeP). The *solid line* indicates the Bonferroni threshold for multiple test correction with a type I error of 5% (*P* value <5.48 × 10^−7^)

We conducted the first GWAS using PLINK v1.07 software [13]. To control the inflation of *P* values due to the confounding with population structure of the lean and fat lines, we tested several models with different covariates in PLINK. The first model fitted line (lean or fat) as a covariate in PLINK. We also carried out principal component analysis (PCA) on the SNPs. The first principal component (PC) clearly separated the two lines. The second PC highlighted genetic differences among individuals in the lean line and the third PC highlighted genetic differences among individuals in the fat line (Fig. 2c, d). Therefore, the second and third models fitted the first two and the first three PC as covariates, respectively, in PLINK. All three models generated similar results. The results obtained by using the first three PC for TeW and TeP are in Fig. 3.

Both Manhattan plots and *Q*–*Q* plots revealed that the *P* values generated by PLINK were inflated. Therefore, we incorporated kinship among individuals in mixed linear models (MLM) to control the inflation of *P* values, using four software packages: (1) GAPIT (version 1) [16], (2) EMMAX (beta) [17], (3) GRAMMAR [18], and (4) GEMMA (version 0.94) [19]. As expected, the mixed linear models (MLM) controlled the inflation of *P* values well. More than 97% of the SNPs were not associated with the two measured traits, TeW and TeP [see Additional file 4: Figure S1]. Based on a Bonferroni threshold (*P* value <5.48 × 10^−7^) for multiple-test correction with a type I error of 5%, 18 and 23 SNPs were found to be significantly associated with TeW and TeP, respectively. Details of these SNPs are in Table 2. They were mainly located on two peaks, one on chicken chromosome 3 and one on chromosome 10. These two peaks were consistent across all analyses. The region on chromosome 3 was 5.63 Mb long (between 59,757,561 and 65,388,568 bp based on the location of the SNPs) and the region on chromosome 10 was 4.72 Mb long (between 8,478,019 and 13,195,918 bp based on the location of the SNPs).

**Table 2:** SNPs in candidate genes significantly associated with TeW and TeP

SNP	Chr	Position	*P* value (TeW)	*P* value (TeP)	Candidate gene	Gene position (Mb)
Gga_rs14364352	3	59,757,561	5.13E−07	1.81E−07	*MAP7*	56.99–57.10
					*TCF21*	58.22–58.22
					*EPB41L2*	59.61–59.68
Gga_rs14364587	3	60,099,912	8.98E−08	1.15E−08	–	–
Gga_rs14705827	3	60,784,548	3.22E−07	3.73E−07	–	–
GGaluGA224607	3	61,317,809	3.66E−07	4.53E−07	–	–
Gga_rs14365191	3	61,522,182	2.25E−07	3.66E−07	–	–
GGaluGA224679	3	61,526,696	1.33E−07	2.42E−07	–	–
Gga_rs16286755	3	64020868	2.58E−08	4.14E−08	*GJA1*	64.41–64.42
Gga_rs16287113	3	64,522,837	2.26E−07	9.13E−07	–	–
GGaluGA225462	3	65,388,568	1.61E−07	3.83E−07	*GPRC6A*	66.36–66.37
Gga_rs14748077	10	8,478,019	9.78E−07	3.53E−07	–	–
GGaluGA068209	10	8,928,839	5.14E−07	1.68E−07	*TEX9*	8.70–8.75
GGaluGA068594	10	10,330,735	5.89E−07	3.80E−07	–	–
GGaluGA068660	10	10,573,304	3.95E−08	1.70E−08	*CYP19A1*	10.55–10.57
GGaluGA068662	10	10,607,832	3.66E−08	7.77E−09	–	–
Gga_rs14703430	10	10,647,843	2.90E−10	4.89E−11	–	–
Gga_rs14005067	10	10,742,112	1.91E−06	5.06E−07	–	–
Gga_rs14946630	10	10,792,181	1.92E−10	2.11E−11	–	–
Gga_rs15575830	10	11,983,111	1.33E−06	4.90E−07	–	–
Gga_rs15575901	10	12,159,992	6.91E−07	2.48E−07	–	–
Gga_rs14947056	10	12,323,517	6.91E−07	2.48E−07	–	–
Gga_rs14005930	10	12,487,480	1.55E−08	4.47E−09	*PDE8A*	12.64–12.75
Gga_rs14006710	10	13,195,918	9.67E−08	1.72E−08	*SH3GL3*	13.10–13.15

Associated SNPs were determined by GWAS on testis weight (TeW) and testis percentage (TeP). GWAS were performed using PLINK with three principal components as covariates to control for population structure

### Identification of candidate genes

Nearby (about 2 Mb region) and within these two regions of interest on chromosomes 3 and 10, 85 and 62 annotated genes were present in the Ensemble database, respectively. Based on database mining and literature searches, we identified nine candidate genes with known functions that are directly related to testis growth and development. These nine genes are *MAP7* (*microtubule*-*associated protein 7*), *TCF21* (*transcription factor 21*), *EPB41L2* (*erythrocyte membrane protein band 4.1*-*like 2*), *GJA1* (*gap junction protein, alpha 1*), *GPRC6A* (*G protein*-*coupled receptor, family C, group 6, member A*), *TEX9* (*testis expressed 9*), *CYP19A1* (*cytochrome P450, family 19, subfamily A, polypeptide 1*), *PDE8A* (*phosphodiesterase 8A*), and *SH3GL3* (*SH3*-*domain GRB2*-*like 3*).

### Analysis of mRNA expression for the nine candidate genes

To investigate the roles of these nine genes, we used real-time quantitative (RT-PCR) to measure their mRNA expression levels in the testis tissue of NEAUHLF individuals. For this analysis, we randomly chose 20 birds from each line in G_16_. *GAPDH* and *TBP* were used as two internal reference genes.

**Fig. 4:**
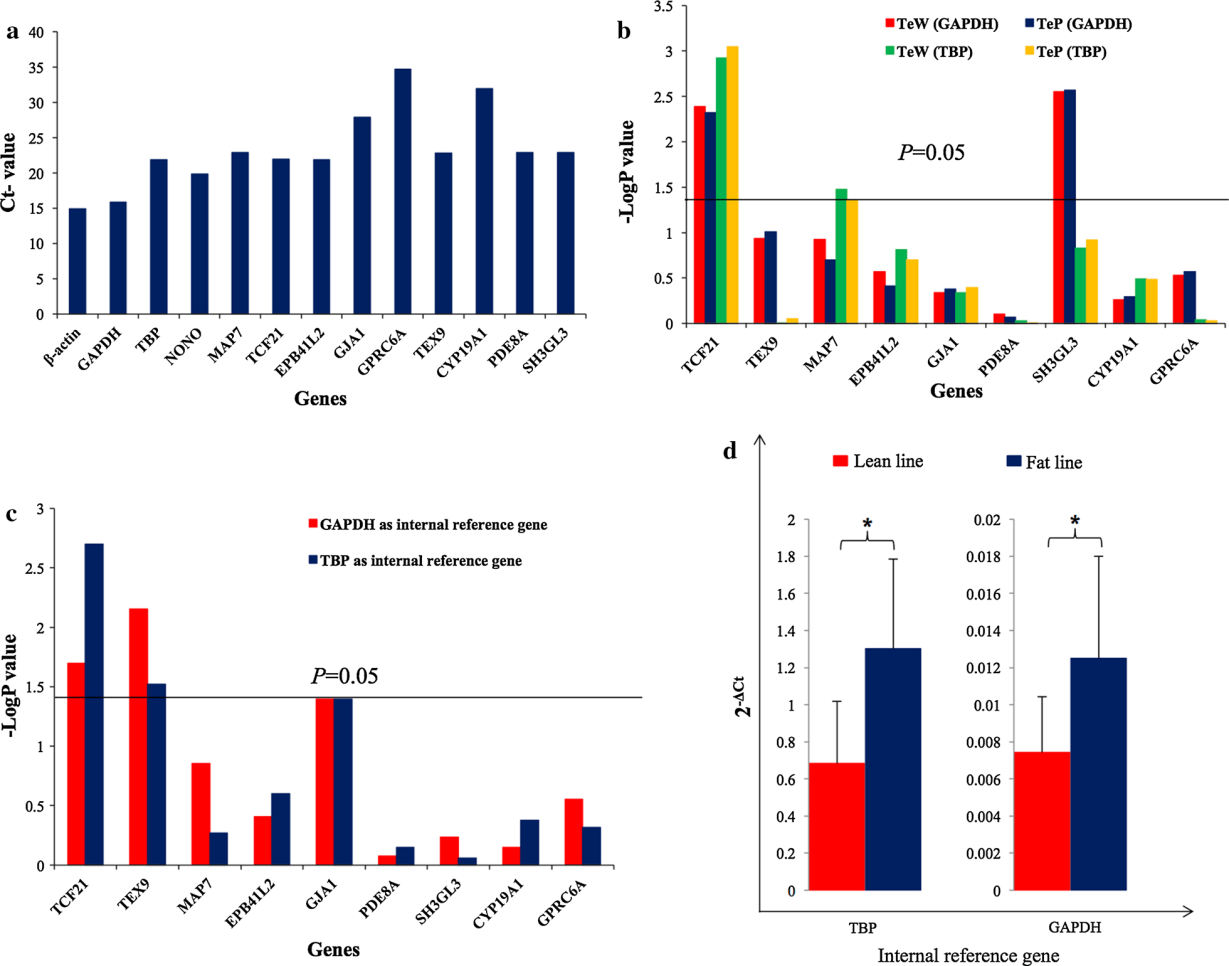
mRNA expression analysis of the nine candidate genes (identified by GWAS) in male broiler testis tissue. **a** *C*_t_ values for the nine candidate genes and four housekeeping genes. **b** –log *P* value of the association between the expression levels of the nine genes and TeW and TeP of birds from the G_16_ population, using two different housekeeping genes as an internal reference. **c** –log *P* value of the difference in expression levels across the nine candidate genes between the lean and fat lines of G_16_, using two different housekeeping genes as an internal reference. **d** Difference in *TCF21* expression level ($2^{{ -\Delta C{\text{t}} }}$ + SE) between the *lean* and *fat lines* at G_19_, using two different housekeeping genes as an internal reference. *P < 0.05, *TeW* testis weight, *TeP* testis percentage

First, we determined the expression level of the nine genes in testis tissue. Using pooled cDNAs of all 40 individuals across the two lines, we found that the expression levels of two genes (*GPRC6A* and *CYP19A1*) were lower than those of the other seven genes. However, all nine genes were expressed in the testis tissue (Fig. 4a), which suggests that they may all play roles in the growth and development of the testis.

Associations between the mRNA expression levels of each of these nine genes with TeW and TeP for the 40 birds were further analyzed. Using both *GAPDH* and *TBP* as internal reference genes, only the mRNA expression level of *TCF21* was significantly correlated (*P* < 0.05) with TeW and TeP (Fig. 4b). We also compared the mRNA expression levels of these nine genes between the two lines. The results indicated that the mRNA expression levels of *TCF21*, *TEX9,* and *GJA1* differed significantly (*P* < 0.05) between the lean and fat lines (Fig. 4c). However, only the expression level of *TCF21* was significantly associated (*P* < 0.05) with TeW and TeP, and significantly different (*P* < 0.05) between the two lines.

### Additional validation on *TCF21*

To further confirm the role of the *TCF21* gene across generations, we conducted a second mRNA expression analysis of *TCF21* using 15 individuals from each line that were randomly chosen from G_19_. The expression level of the *TCF21* gene in the lean line was again significantly lower (*P* < 0.05) than that in the fat line (Fig. 4d and Figure S2 [see Additional file 5: Figure S2]. This finding confirmed the results obtained on the G_16_ birds. Combined together, these results suggest that *TCF21* is a major gene involved in testis growth and development in chickens.

**Fig. 5:**
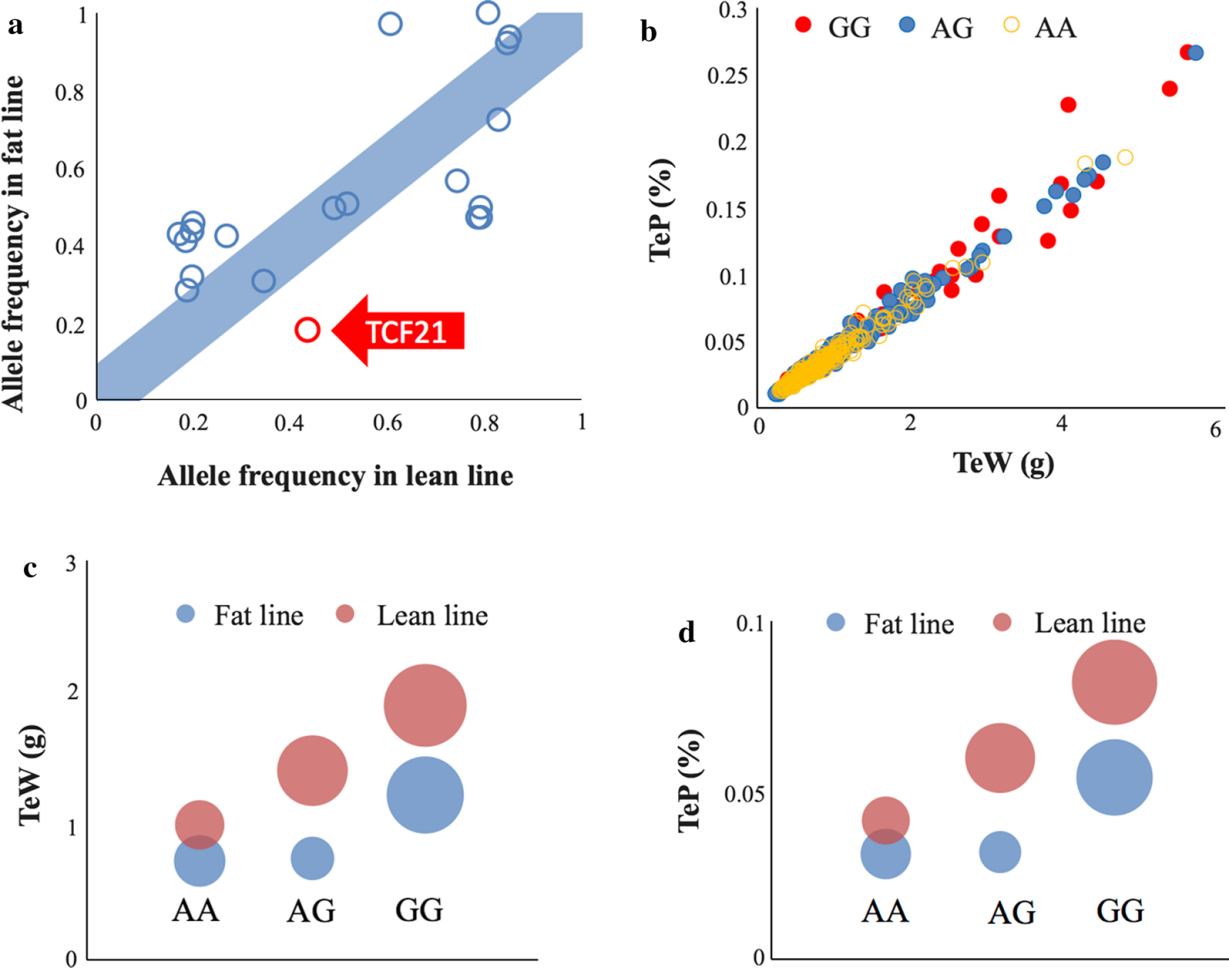
Allele frequencies of the significant SNPs that are linked to the *TCF21* gene. Allele frequencies of the significant SNPs within the regions of interest on chromosomes 3 and 10 were calculated for both *lean* and *fat lines*. When plotted against each other, SNPs near the diagonal demonstrated similar frequencies between the* two lines*. The SNP associated with *TCF21* is one of the SNPs that deviates from the diagonal (**a**). When the individuals were characterized by the genotypes associated with the *TCF21* SNP (Gga_rs14364352), individuals with genotypes *AG* (170 birds) and *GG* (52 birds) appear to have larger values for both TeW and TeP than those with *AA* (253 birds) genotype (**b**). The individuals with *AG* and *GG* genotypes have higher values than individuals with *AA* genotypes for both TeW (**c**) and TeP (**d**). This trend was the same in both* lean* and* fat lines*. The individuals with *AG* and *GG* genotypes also have larger variations in trait values than individuals with *AA* genotypes. Standard error of the mean is indicated by the size of the* circles*

We also observed that the frequencies of the allele (*G*) of the *TCF21*-associated SNP (Gga_rs14364352) with higher TeW and TeP differed between the lean line (43.6%) and the fat line (17.8%) in generation 11. More importantly, differences in testis traits between genotypes at this SNP displayed similar trends in the lean and fat lines, which means that, in both lines, *GG* is always associated with higher TeW and TeP than *AA* and *AG* (Fig. 5). The *GG* genotype exhibited an additive effect in the lean line. The individuals with the *GG* genotype had testis pairs that weighed about one gram more than individuals with the *AA* genotype, whereas individuals with the *AG* genotype had intermediate values. The genotypes of the *TCF21*-associated SNP had a dominant effect in the fat line, with *A* being the recessive allele. Consequently, the TeW of individuals with *AA* or *AG* genotypes were similar and were about 0.5 g less than the TeW of individuals with the *GG* genotype.

## Discussion

### Differences in testis size between the lean and fat lines

The lean and fat broiler lines used in the current study were selected for and against abdominal fat content over 19 generations. Abdominal fat percentages were significantly different (*P* < 0.05) between the lean and fat lines from G_4_ to G_19_, while no significant differences (*P* > 0.05) were observed in BW7 (Fig. 1a, b). Therefore, these two lines were ideal populations to study the genetic mechanisms that underlie abdominal fat deposition.

Selection for abdominal fat content was the primary objective of the two divergently selected lines. We observed that birds from the fat line had a significantly smaller (*P* < 0.05) testis size (TeW and TeP) in early growth than birds from the lean line (Fig. 1c, d). Cellular development at 6 to 7 weeks of age determines the fertility of male broiler breeders [22]. Thus, 7-week-old broilers, as used in our study, reflect the fertility of adults.

Testis size is controlled by genetic factors in both chicken and mice [22–26]. Roosters with small testes often have poor fertility [9]. Similar to findings in chicken, studies in humans have shown that obese men have an increased incidence of low fertility, with reduced serum levels of androgens and sex hormone-binding globulin (SHBG) and increased levels of estrogen [4].

### Identification of candidate genes for testis growth and development

Using GWAS, two regions, i.e. one on chicken chromosome 3 (between 59.8 and 65.4 Mb) and one on chromosome 10 (between 8.5 and 13.2 Mb) were detected and found to be significantly associated (*P* < 0.05) with TeW and TeP (Fig. 3). These two regions were consistently detected with PLINK, as well as with several other software packages: GAPIT, EMMAX, GenABEL (GRAMMAR) and GEMMA [see Additional file 4: Figure S1]. This substantial concordance was interesting, although the *P* values from the four latter analyses were less significant than those from PLINK [13]. These results indicate that these two regions may harbor genes that play a role in testis growth and development. Unfortunately, we found no QTL for reproduction performance of male broilers in these regions in the animal QTL database (http://www.animalgenome.org/cgi-bin/QTLdb/index).

We catalogued all genes that were located nearby and within these two regions on chromosomes 3 and 10, and identified 85 and 62 genes, respectively. Based on the known functions of these genes, we identified nine candidate genes: *MAP7*, *TCF21*, *EPB41L2*, *GJA1*, *GPRC6A*, *TEX9*, *CYP19A1*, *PDE8A* and *SH3GL3*, which may be involved in testis growth and development.

### The *TCF21* gene is important for testis growth and development in broiler chickens

To further investigate these nine candidate genes, we analyzed their mRNA expression levels. For all nine genes, we calculated the following: (1) associations of their mRNA expression levels with TeW and TeP, and (2) differences in mRNA expression levels between the two lines. We found that *TCF21* was the only gene that had both an mRNA expression level that was significantly associated (*P* < 0.05) with TeW and TeP and that differed significantly (*P* < 0.05) between the two lines. This result was found for both G_16_ and G_19_ birds, i.e. birds from the fat line consistently exhibited significantly higher (*P* < 0.05) *TCF21* expression levels than birds from the lean line.

Frequencies of allele *G* at the most significant SNP (Gga_rs14364352) near *TCF21*, which is associated with higher testicular weight, differed substantially between the fat (17.8%) and lean (43.6%) lines. Because the number of individuals with all three genotypes in the lean and fat lines respectively was too small, we did not carry out an analysis of *TCF21* expression between different genotypes.

To the best of our knowledge, there is only one article in the literature on the function of *TCF21* in chickens, which showed that *TCF21* can inhibit the differentiation of epicardium-derived cells into smooth muscle in the developing heart [27]. In chickens, humans, and mice, TCF21 protein sequences share a high percentage of sequence similarity (91 to 92%) [see Additional file 6: Table S4], which suggests that the chicken *TCF21* gene may have similar functions as the *TCF21* gene in humans and mice. In humans and mice, *TCF21* is known to play an important role in diseases such as hypertension, gastric cancer, and coronary heart disease [28–30]. In mice, *TCF21* is also the first direct downstream target of the male sex-determining factor, *SRY* [31, 32], and the knockout of *TCF21* results in male-to-female sex reversal in mice [33]. *SRY* binds to the *TCF21* promoter and activates gene expression [32]. *TCF21* and *SRY* also have similar effects on Sertoli cell differentiation and embryonic testis development in rats [32]. Taken together, these results suggest that the *TCF21* gene may play an important role in sex differentiation and testis development in chicken. In addition, our study showed that the birds from the lean line, with higher TeW and TeP, had significantly lower (*P* < 0.05) *TCF21* expression levels than birds from the fat line.

It is interesting to note that, in chickens, the relationship between *TCF21* expression and testis size is opposite to that observed in mice and rats, i.e. birds from the fat line had higher *TCF21* expression levels and smaller testes, whereas in mice and rats *TCF21* gene is a positive transcriptional regulation factor. In addition, in mice and rats *SRY* binds to the *TCF21* promoter and activates gene expression [32] but the chicken genome contains no *SRY* gene. Thus, our findings indicate that the *TCF21* gene may affect testis growth and development in chickens through a different mechanism than in mice or rats.

It has also been shown that the *TCF21* gene (1) is expressed in white but not brown adipocytes, which means that *TCF21* is specific to white fat [34–36], and (2) can positively regulate the expression of bone morphogenetic protein 4 (*BMP4*). *BMP4* can commit pluripotent mesenchymal cells to form white adipocytes [37], which suggests that *TCF21* may have a potential role in adipogenesis.

Studies on humans and mice indicated that *TCF21* may play an important role in both adipocyte development and testis growth, and thus, these two processes may be closely related. In our study, we found that chickens with high AFW (or AFP) often had low TeW (or TeP) (Table 1), which agrees with findings in humans and mice. Overall, these results suggest that the chicken *TCF21* may not only be important for testis growth, but also play a role in abdominal fat deposition but further studies are required for confirmation.

A previous selective sweep analysis using the same dataset [38] did not find a significant signal for the coding region of *TCF21* (at the position of 58.22 Mb on chromosome 3). However, a nearby region (59.3–64.7 Mb) contained seven core haplotypes with a relative extended haplotype homozygosity (REHH) *P* value <0.05. Similarly, we also detected a region (between 8.7 and 13.3 Mb) on chromosome 10 that contained 14 core haplotypes with a REHH *P* value <0.05 [see Additional file 7: Table S5]. Thus, based on the results of the selective sweep analysis, the two regions on chromosomes 3 and 10 may harbor important genes for testis growth.

In addition to *TCF21,* the other eight candidate genes selected based on the GWAS also have known functions associated with reproductive and fat traits [see Additional file 8: Supplementary text]. However, together with the expression results of these genes, which showed that *TCF21* was the only gene that was differently expressed between the two lines and significantly associated with TeW and TeP, we focused on *TCF21* as an important gene for testis growth and development in broiler chickens.

## Conclusions

Two chromosomal regions in the chicken, a 5.63 Mb region on chromosome 3 and a 4.72 Mb region on chromosome 10, were found to be associated with testis growth and development in broiler chickens. We identified nine genes within these regions that had functions related to testis growth and development: *MAP7*, *TCF21*, *EPB41L2*, *GJA1*, *GPRC6A*, *TEX9*, *CYP19A1*, *PDE8A,* and *SH3GL3*. Using two independent mRNA expression experiments, one of these nine genes, *TCF21*, was confirmed to be significantly associated with testis weight and testis percentage.

## Additional files

**Additional file 1: Table S1.** Generations of NEAUHLF chickens used for the different analyses in the current study.

**Additional file 2: Table S2.** Number of SNPs and average density on each chromosome.

**Additional file 3: Table S3.** Primer sequences used to analyze gene expression.

**Additional file 4: Figure S1.** Manhattan and *Q*–*Q* plots of genome-wide association analyses for TeW and TeP, generated with GAPIT, EMMAX, GenABEL (GRAMMAR) and GEMMA software. The solid line indicates genome-wide significance of association (*P* value <5.48 × 10^−7^). *TeW* testis weight, *TeP* testis percentage.

**Additional file 5: Figure S2.** Scatter plots of the expression level ($2^{{ -\Delta C_{\text{t}} }}$) of the *TCF21* gene in testis tissue versus testis weight (TeW) in the lean and fat lines from G_19_ using two different housekeeping genes as internal references. TeW = testis weight.

**Additional file 6: Table S4.** Alignment of chicken TCF21 protein sequences with those of other species.

**Additional file 7: Table S5.** Summary of statistics for core haplotypes with *P* < 0.05 on chromosome 3 between 59.3 and 64.7 Mb and on chromosome 10 between 8.7 and 13.3 Mb after the relative extended haplotype homozygosity (REHH) test.

**Additional file 8** Known functions of the remaining eight candidate genes. Description: This file describes the basic functions of the other eight genes detected in the GWAS and their possible involvement in spermatogenesis [39–82].
