# Supplementary material for: TCF21 is related to testis growth and development in broiler chickens
Source: Genet Sel Evol. 2017 Feb 24;49:25. doi: 10.1186/s12711-017-0299-0 (PMC5326497; doi:10.1186/s12711-017-0299-0)
Supplement: Supplementary file 1 — Additional file 1: Table S1. Generations of NEAUHLF chickens used for the different analyses in the current study. [file 12711_2017_299_MOESM1_ESM.doc]

**Additional file 1: Table S1.**

**Table S1. Generations of NEAUHLF chickens used for the different analyses in the current study.**

| Generations | Number of birds | Used in the differences analyses of TeW and TeP | Used in the GWAS | Used in the mRNA expression validation analysis |
| --- | --- | --- | --- | --- |
| G_7_ | 472 | √ |  |  |
| G_8_ | 387 | √ |  |  |
| G_9_ | 383 | √ |  |  |
| G_10_ | 627 | √ |  |  |
| G_11_ | 475 | √ | √ |  |
| G_12_ | 521 | √ |  |  |
| G_13_ | 598 | √ |  |  |
| G_14_ | 612 | √ |  |  |
| G_15_ | 538 | √ |  |  |
| G_16_ | 665 | √ |  | √ |
| G_17_ | 627 | √ |  |  |
| G_18_ | 583 | √ |  |  |
| G_19_ | 509 | √ |  | √ |
