# Supplementary material for: TCF21 is related to testis growth and development in broiler chickens
Source: Genet Sel Evol. 2017 Feb 24;49:25. doi: 10.1186/s12711-017-0299-0 (PMC5326497; doi:10.1186/s12711-017-0299-0)
Supplement: Supplementary file 2 — Additional file 2: Table S2. Number of SNPs and average density on each chromosome. [file 12711_2017_299_MOESM2_ESM.doc]

**Additional file 2: Table S2.**

**Table S2. Number of SNPs** and average density on each chromosome

| Chromosome | Number of SNPs | Density (kb/SNP) | Chromosome | Number. of SNPs | Density (kb/SNP) |
| --- | --- | --- | --- | --- | --- |
| 1 | 7143 | 28.136 | 18 | 845 | 12.898 |
| 2 | 5299 | 29.217 | 19 | 804 | 12.321 |
| 3 | 4081 | 27.855 | 20 | 1460 | 9.541 |
| 4 | 3314 | 28.423 | 21 | 726 | 9.483 |
| 5 | 2172 | 28.662 | 22 | 295 | 13.234 |
| 6 | 1714 | 20.920 | 23 | 577 | 10.456 |
| 7 | 1770 | 21.576 | 24 | 676 | 9.229 |
| 8 | 1394 | 21.985 | 25 | 170 | 11.930 |
| 9 | 1168 | 20.585 | 26 | 617 | 8.169 |
| 10 | 1297 | 17.300 | 27 | 472 | 10.271 |
| 11 | 1196 | 18.302 | 28 | 563 | 7.932 |
| 12 | 1324 | 15.455 | LGE22^A^ | 103 | 8.651 |
| 13 | 1128 | 16.253 | LGE64 | 2 | 2.289 |
| 14 | 984 | 16.036 | Z | 1844 | 40.472 |
| 15 | 1010 | 12.810 | UN^B^ | 606 | UN |
| 16 | 13 | 34.823 | Total | 45611 | 22.511 |
| 17 | 844 | 12.590 | / | / | / |

^A^LGE22 = LGE22C19W28_E50C23

^B^UN, unknown chromosome and position
