# Supplementary material for: TCF21 is related to testis growth and development in broiler chickens
Source: Genet Sel Evol. 2017 Feb 24;49:25. doi: 10.1186/s12711-017-0299-0 (PMC5326497; doi:10.1186/s12711-017-0299-0)
Supplement: Supplementary file 3 — Additional file 3: Table S3. Primer sequences used to analyze gene expression. [file 12711_2017_299_MOESM3_ESM.doc]

## Additional file 3: Table S3.

## Table S3. Primer sequences used to analyze gene expression

| *Gene* | Accession number | Primer sequence 5ʹ to 3ʹ |
| --- | --- | --- |
| *GAPDH* | NM_204305 | F:AGAACATCATCCCAGCGT  R:AGCCTTCACTACCCTCTTG |
| *TBP* | NM_205103 | F:GCGTTTTGCTGCTGTTATTATGAG  R:TCCTTGCTGCCAGTCTGGAC |
| *MAP7* | NM_001031072 | F:CCTGGTTCCTCCAAAGTTCC  R:GATGGGCTGGCTGCTTG |
| *TCF21* | NM_001277711 | F:ACGCTGCCAACGCAAGGG  R:TGTTCACCACTTCTTTCAGGTCACTC |
| *EPB41L2* | XM_015284141 | F:GGAATCCACACCCGAACCACG  R:TCTTCTATCCCAACCTCTGCCTGCT |
| *GJA1* | NM_204586 | F: CCACTCGCACTGCTCAT  R:TCCACGCATCTTTACCTT |
| *GPRC6A* | XM_426177 | F:GAGGTGTCAATGGCAGTT  R:GTCATCATCTGTGGCTATTAC |
| *TEX9* | XM_015291872 | F:GAAGAGCTGGATAGCATCATGTGTG  R:AACAACCTCTTGCTGTAATCCTTCG |
| *CYP19A1* | NM_001001761 | F:TGTTCCATCACGCTATTT  R:GATTCTTGTTTGGGCTTC |
| *PDE8A* | XM_004943816 | F:GCTCACAAAGGCGGCACTCTTCTAT  R:CCTGTGCTATCCGTGGGGGTATG |
| *SH3GL3* | NM_204528 | F:CACAGAGTATCTTCAGCCAAAT  R:GCCAAACATAGAATCATCG |
