# Supplementary material for: TCF21 is related to testis growth and development in broiler chickens
Source: Genet Sel Evol. 2017 Feb 24;49:25. doi: 10.1186/s12711-017-0299-0 (PMC5326497; doi:10.1186/s12711-017-0299-0)

**Additional File 4: Figure S1.**

**Figure S1.** Manhattan and Q-Q plots of genome-wide association analyses for TeW and TeP generated with GAPIT, EMMAX, GenABEL (GRAMMAR) and GEMMA software.

The solid line indicates genome-wide significance of association (*P*-value < 5.48 × 10^−7^). TeW = testis weight; TeP = testis percentage.


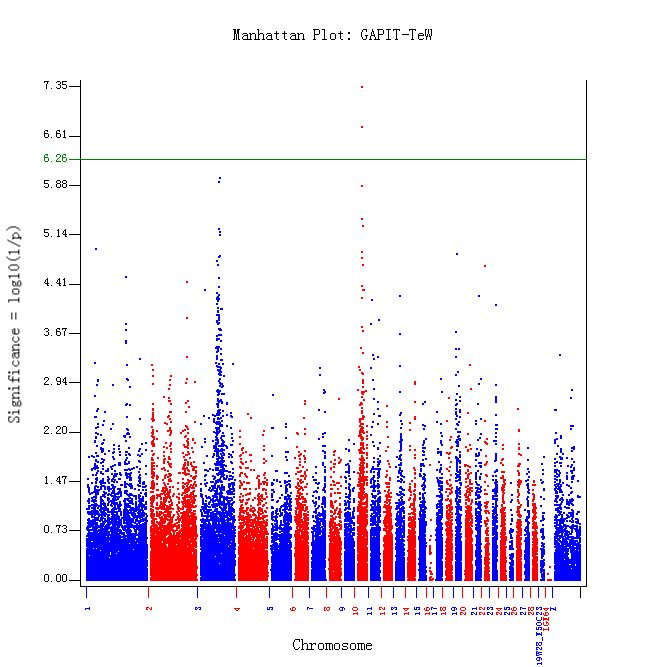


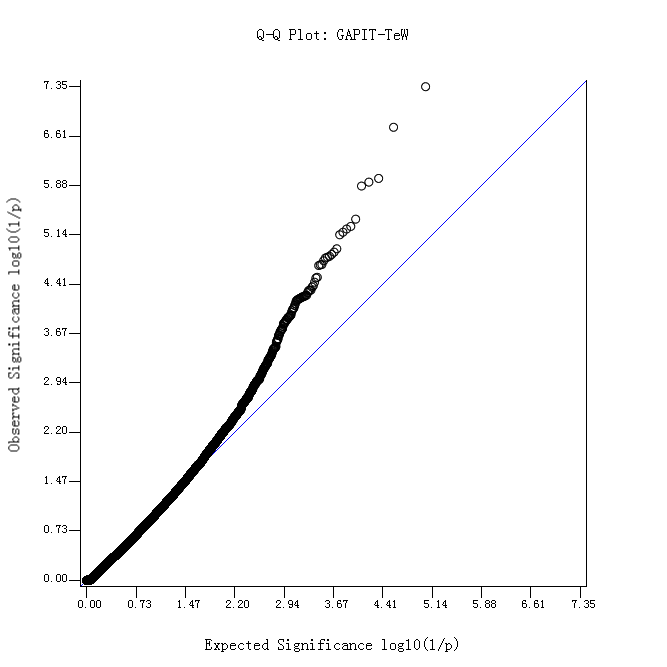


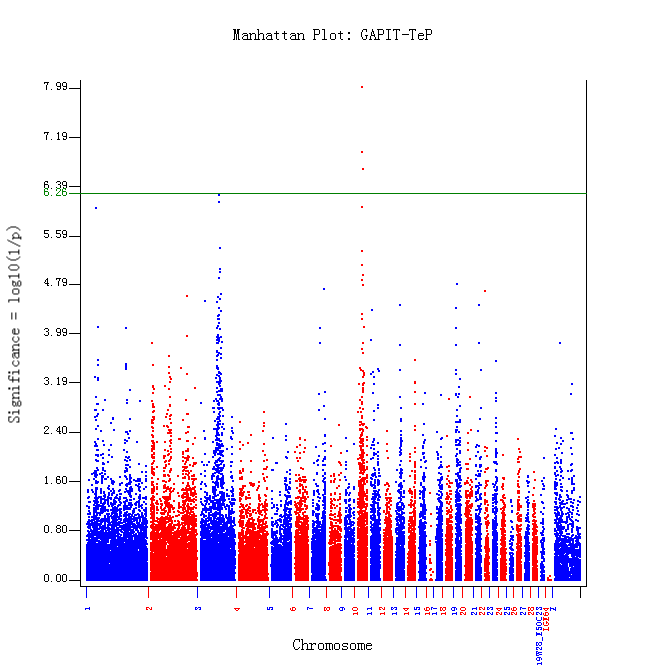


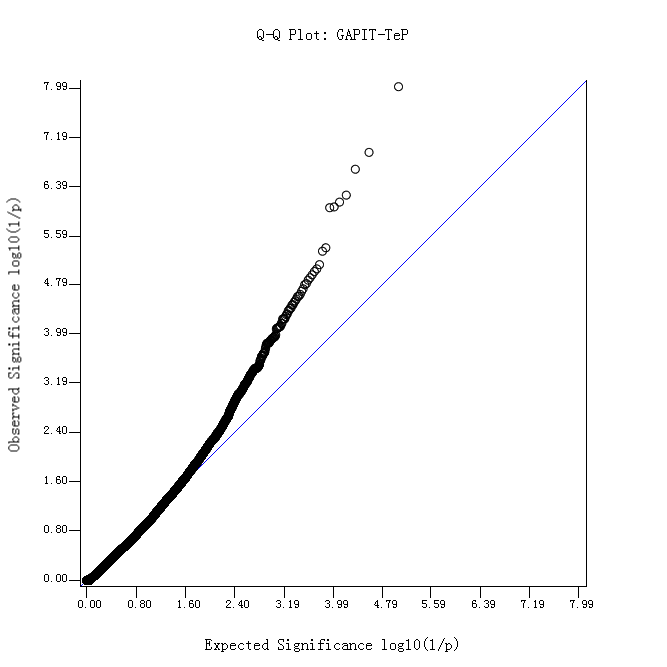


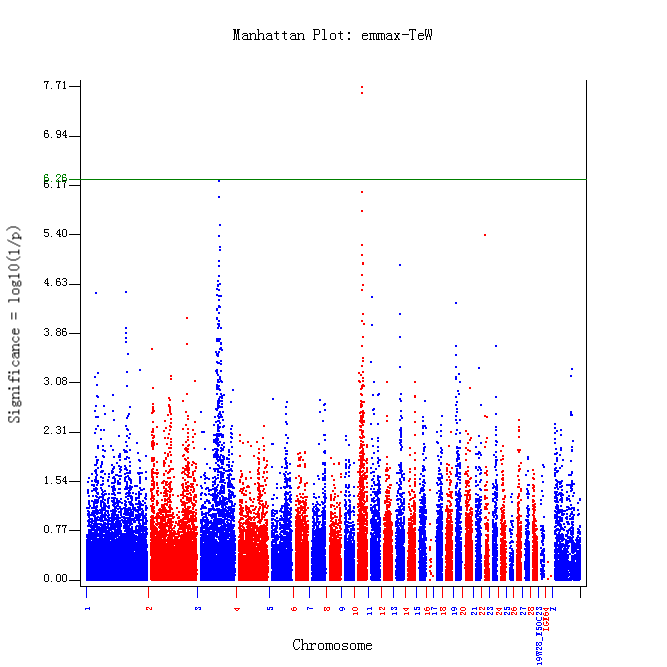


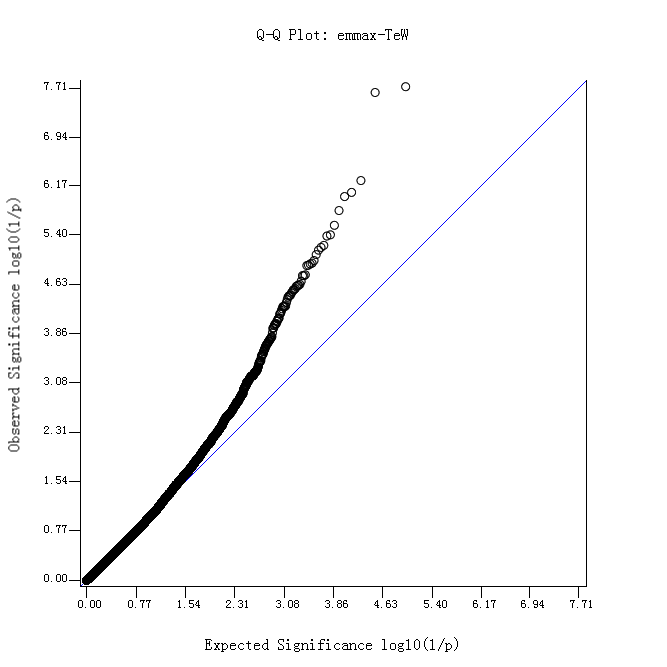


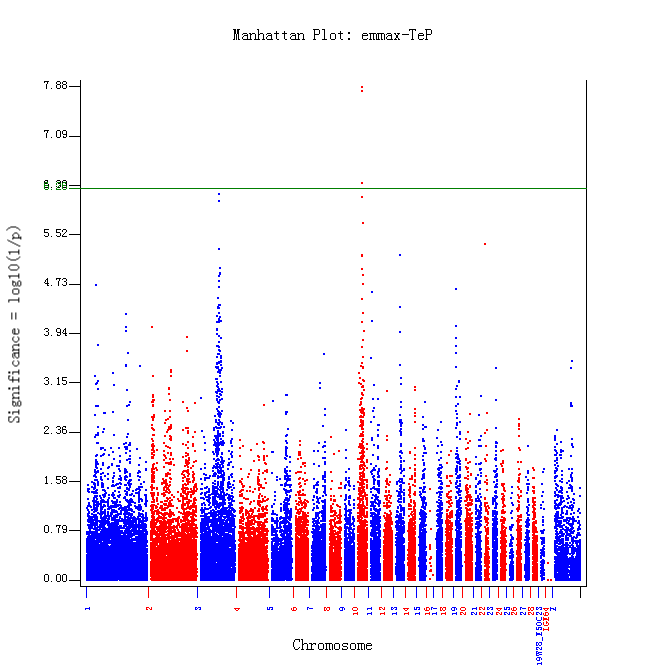


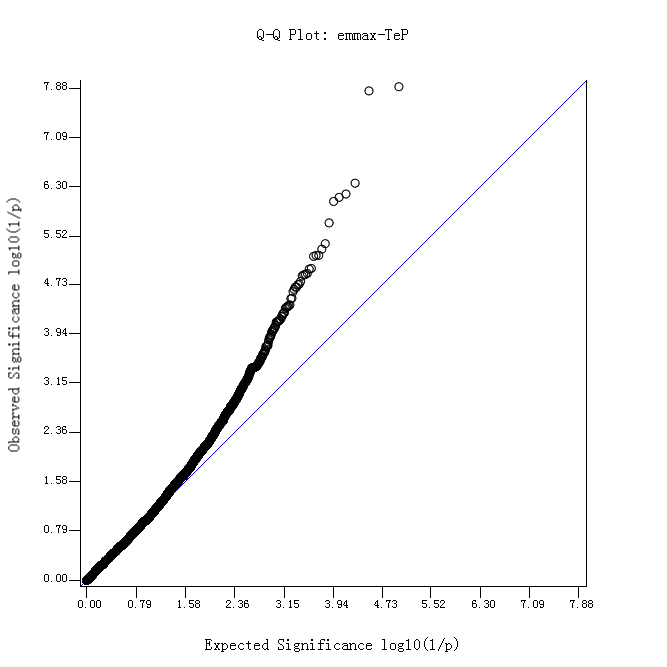


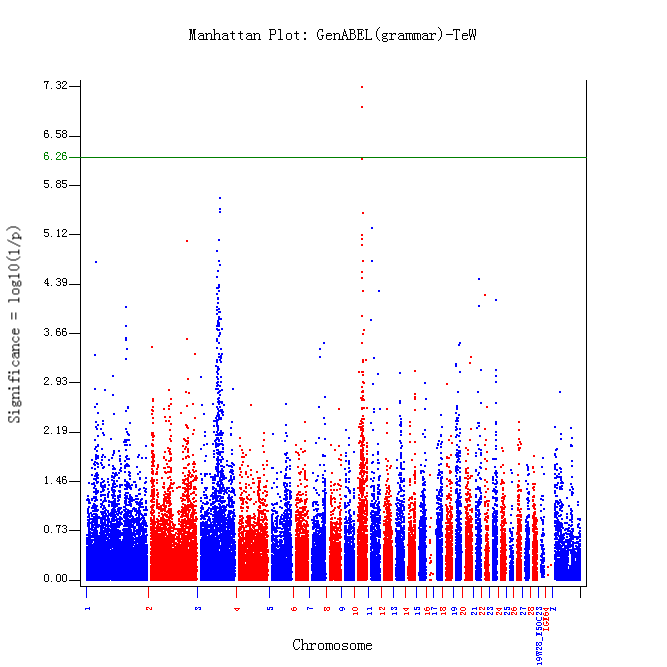


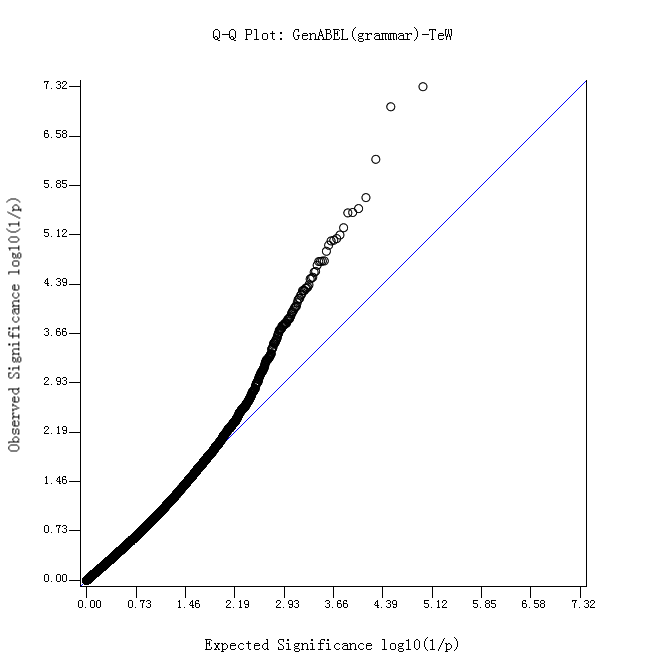


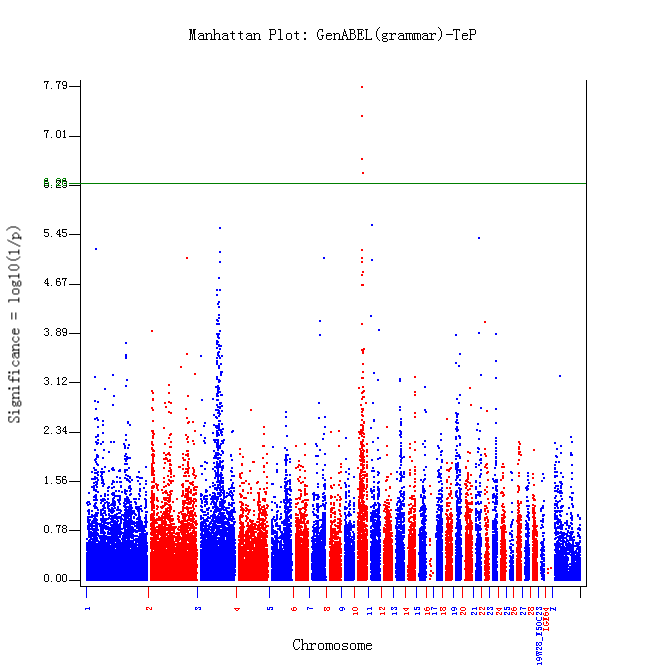


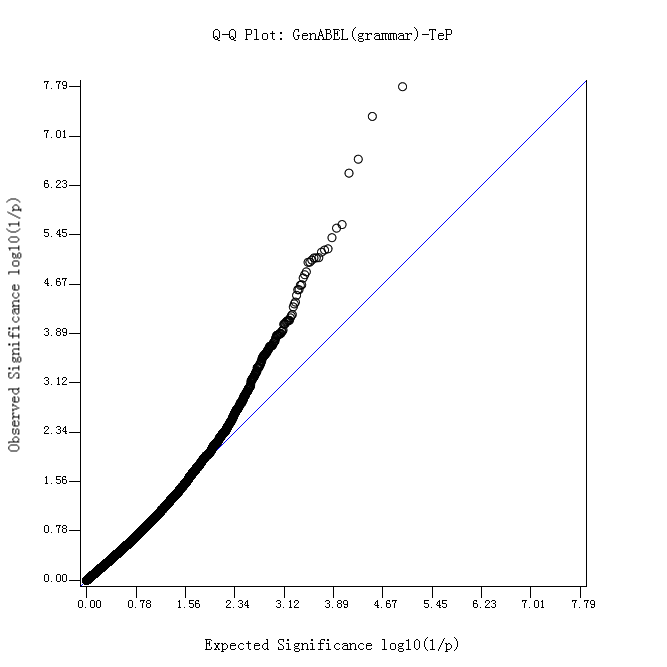


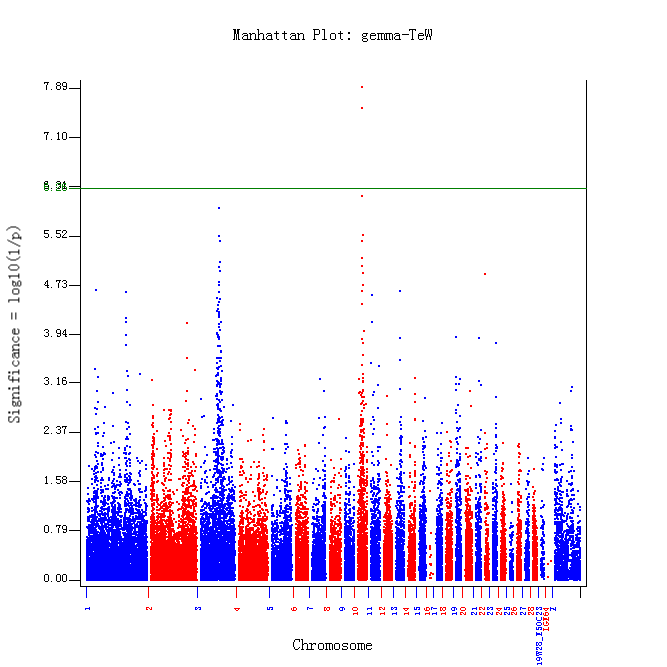


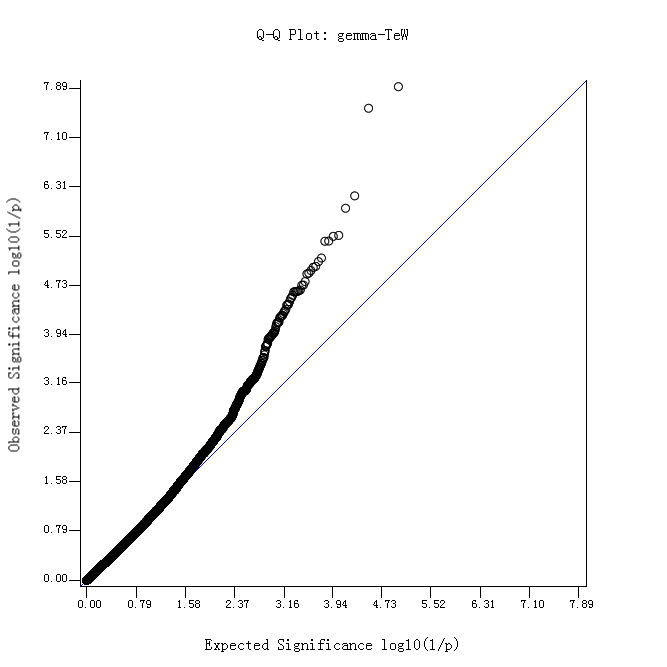


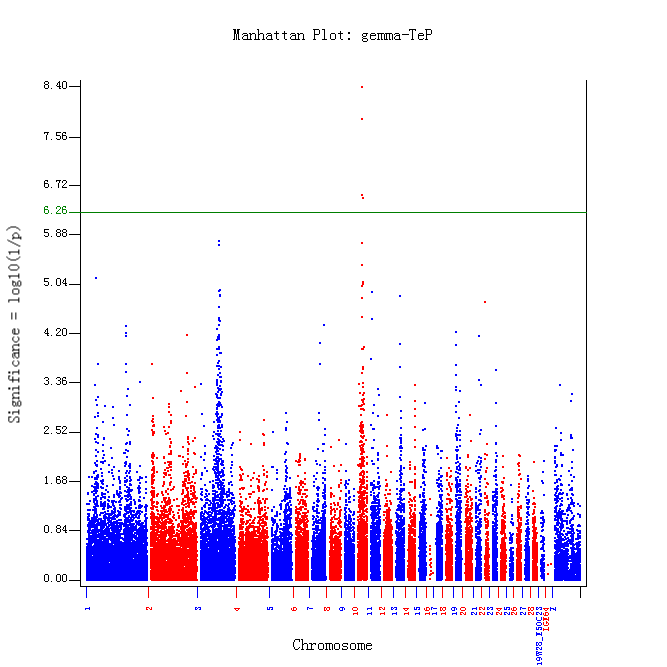


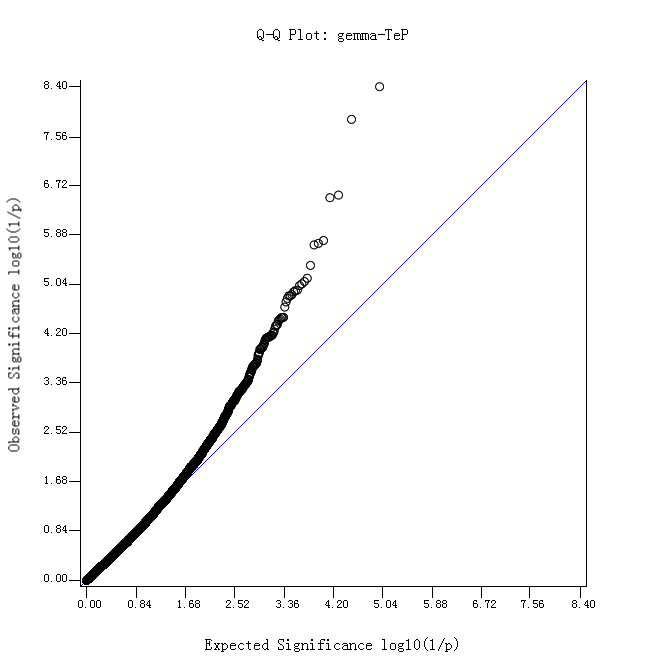

Supplement: Supplementary file 4 — Additional file 4: Figure S1. Manhattan and Q–Q plots of genome-wide association analyses for TeW and TeP, generated with GAPIT, EMMAX, GenABEL (GRAMMAR) and GEMMA software. The solid line indicates genome-wide significance of association (P value <5.48 × 10^−7^). TeW testis weight, TeP testis percentage. [file 12711_2017_299_MOESM4_ESM.doc]
