# Supplementary material for: TCF21 is related to testis growth and development in broiler chickens
Source: Genet Sel Evol. 2017 Feb 24;49:25. doi: 10.1186/s12711-017-0299-0 (PMC5326497; doi:10.1186/s12711-017-0299-0)

**Additional File 5: Figure S2.**

**Figure S2.** Scatter plots of the expression level (2^-ΔCt^) of the *TCF21* gene in testis tissue *vs.* testis weight (TeW) in the lean and fat lines from G_19_ using two different housekeeping genes as internal references.


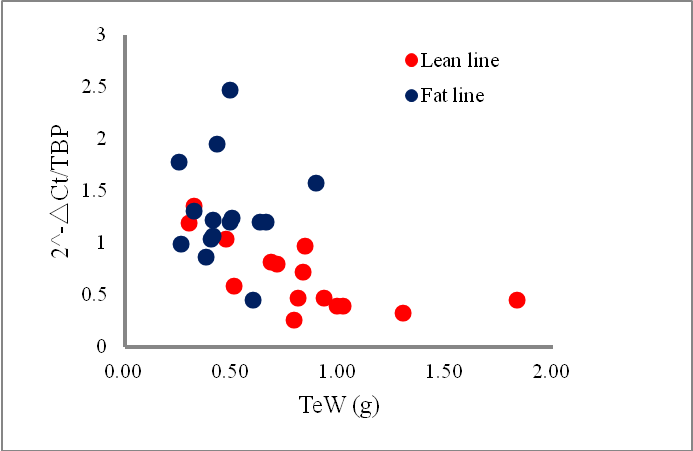


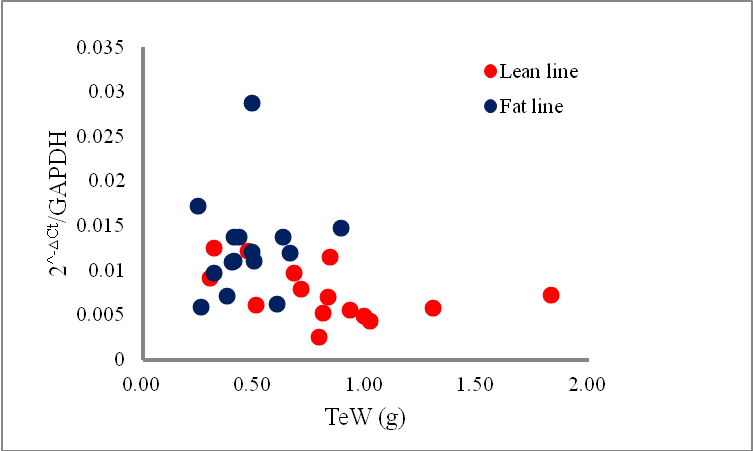

Supplement: Supplementary file 5 — Additional file 5: Figure S2. Scatter plots of the expression level ($2^{{ -\Delta C_{\text{t}} }}$2-ΔCt) of the TCF21 gene in testis tissue versus testis weight (TeW) in the lean and fat lines from G19 using two different housekeeping genes as internal references. TeW = testis weight. [file 12711_2017_299_MOESM5_ESM.doc]
