# Supplementary material for: TCF21 is related to testis growth and development in broiler chickens
Source: Genet Sel Evol. 2017 Feb 24;49:25. doi: 10.1186/s12711-017-0299-0 (PMC5326497; doi:10.1186/s12711-017-0299-0)
Supplement: Supplementary file 6 — Additional file 6: Table S4. Alignment of chicken TCF21 protein sequences with those of other species. [file 12711_2017_299_MOESM6_ESM.doc]

## Additional file 6: Table S4.

## Table S4. Alignment of chicken TCF21 protein sequences with those of other species

| Species | Accession No. | Percentage of sequence similarity |
| --- | --- | --- |
| *Acyrthosiphon pisum* | NP_001156349.1 | 62% |
| *Anas platyrhynchos* | EOB05373.1 | 99% |
| *Apaloderma vittatum* | KFP84583.1 | 99% |
| *Bos taurus* | NP_001014899.1 | 91% |
| *Camelus ferus* | EQB78786.1 | 80% |
| *Caprimulgus carolinensis* | KFZ54636.1 | 99% |
| *Cariama cristata* | KFP57145.1 | 100% |
| *Chaetura pelagica* | KFU90764.1 | 97% |
| *Chelonia mydas* | EMP37394.1 | 96% |
| *Clonorchis sinensis* | GAA53993.1 | 53% |
| *Columba livia* | EMC83324.1 | 100% |
| *Crassostrea gigas* | EKC39937.1 | 78% |
| *Danio rerio* | NP_001032770.1 | 74% |
| *Echinococcus granulosus* | CDS18456.1 | 75% |
| *Echinococcus multilocularis* | CDJ01513.1 | 75% |
| *Egretta garzetta* | KFP20554.1 | 99% |
| *Fulmarus glacialis* | KFV98809.1 | 99% |
| *Gavia stellata* | KFV60413.1 | 93% |
| *Haliaeetus albicilla* | KFP99970.1 | 99% |
| *Homo sapiens* | NP_003197.2 | 92% |
| *Hymenolepis microstoma* | CDS33880.1 | 73% |
| *Lepeophtheirus salmonis* | ACO12725.1 | 41% |
| *Macaca mulatta* | NP_001247887.1 | 91% |
| *Manacus vitellinus* | KFW80941.1 | 99% |
| *Mus musculus* | NP_035675.1 | 91% |
| *Nestor notabilis* | KFQ50401.1 | 99% |
| *Opisthocomus hoazin* | KFR07361.1 | 99% |
| *Phoenicopterus ruber ruber* | KFQ84382.1 | 97% |
| *Rattus norvegicus* | NP_001027569.1 | 91% |
| *Saccoglossus kowalevskii* | NP_001158451.1 | 59% |
| *Salmo salar* | ACI68100.1 | 72% |
| *Schistosoma haematobium* | KGB38030.1 | 77% |
| *Scleropages formosus* | KKW99777.1 | 65% |
| *Stegodyphus mimosarum* | KFM56721.1 | 61% |
| *Tauraco erythrolophus* | KFV08092.1 | 100% |
| *Xenopus (Silurana) tropicalis* | NP_001103518.1 | 88% |
| *Xenopus laevis* | NP_001085957.1 | 87% |
| *Zootermopsis nevadensis* | KDR19329.1 | 67% |
