# Supplementary material for: TCF21 is related to testis growth and development in broiler chickens
Source: Genet Sel Evol. 2017 Feb 24;49:25. doi: 10.1186/s12711-017-0299-0 (PMC5326497; doi:10.1186/s12711-017-0299-0)
Supplement: Supplementary file 7 — Additional file 7: Table S5. Summary of statistics for core haplotypes with P < 0.05 on chromosome 3 between 59.3 and 64.7 Mb and on chromosome 10 between 8.7 and 13.3 Mb after the relative extended haplotype homozygosity (REHH) test. [file 12711_2017_299_MOESM7_ESM.doc]

## Additional file 7: Table S5.

## Table S5. Summary of statistics for core haplotypes with *P* < 0.05 on chromosome 3 between 59.3 and 64.7 Mb and on chromosome 10 between 8.7 and 13.3 Mb after the relative extended haplotype homozygosity (REHH) test.

| Lines | Chr. | Start base | End base | Haplotype frequency | EHH | REHH | REHH *P*-value |
| --- | --- | --- | --- | --- | --- | --- | --- |
| Fat | 3 | 59335394 | 59371814 | 0.26 | 1.00 | 3.20 | 0.0106 |
| Lean | 3 | 60131800 | 60179255 | 0.09 | 1.00 | 3.17 | 0.0423 |
| Lean | 3 | 60672286 | 60755041 | 0.17 | 1.00 | 2.57 | 0.0424 |
| Fat | 3 | 60784548 | 60818185 | 0.15 | 1.00 | 3.11 | 0.0444 |
| Fat | 3 | 61317033 | 61357871 | 0.50 | 0.88 | 2.35 | 0.0455 |
| Fat | 3 | 63526005 | 64020868 | 0.19 | 1.00 | 2.90 | 0.0432 |
| Lean | 3 | 64595403 | 64703200 | 0.45 | 1.00 | 2.97 | 0.0068 |
| Fat | 10 | 8658273 | 8689055 | 0.16 | 1.00 | 2.76 | 0.0437 |
| Fat | 10 | 9551012 | 9794844 | 0.31 | 0.87 | 2.57 | 0.0451 |
| Lean | 10 | 9764180 | 9794844 | 0.17 | 0.97 | 2.99 | 0.0465 |
| Lean | 10 | 9993876 | 10044377 | 0.19 | 0.88 | 3.04 | 0.0423 |
| Lean | 10 | 10372217 | 10479815 | 0.05 | 1.00 | 3.73 | 0.0257 |
| Lean | 10 | 11380407 | 11428996 | 0.24 | 1.00 | 3.17 | 0.0116 |
| Lean | 10 | 11515716 | 11592137 | 0.40 | 0.52 | 2.07 | 0.0226 |
| Lean | 10 | 11684478 | 11710954 | 0.35 | 0.73 | 2.33 | 0.0337 |
| Fat | 10 | 11791682 | 11821372 | 0.28 | 0.91 | 2.86 | 0.0460 |
| Lean | 10 | 12334544 | 12356167 | 0.18 | 0.97 | 4.17 | 0.0064 |
| Lean | 10 | 12815720 | 12845112 | 0.05 | 1.00 | 3.91 | 0.0227 |
| Lean | 10 | 12973366 | 12997673 | 0.41 | 0.41 | 1.91 | 0.0331 |
| Lean | 10 | 13167413 | 13195918 | 0.21 | 1.00 | 3.33 | 0.0087 |
| Fat | 10 | 13243531 | 13265699 | 0.15 | 1.00 | 3.11 | 0.0451 |
