# Supplementary material for: TCF21 is related to testis growth and development in broiler chickens
Source: Genet Sel Evol. 2017 Feb 24;49:25. doi: 10.1186/s12711-017-0299-0 (PMC5326497; doi:10.1186/s12711-017-0299-0)
Supplement: Supplementary file 8 — Additional file 8 Known functions of the remaining eight candidate genes. Description: This file describes the basic functions of the other eight genes detected in the GWAS and their possible involvement in spermatogenesis [39–82]. [file 12711_2017_299_MOESM8_ESM.doc]

# Additional file 8

# Known functions of the remaining eight candidate genes

Microtubules play a key role in spermatogenesis as described in detail by Komada et al. [39]. The *MAP7* gene is important for stabilizing and reorganizing microtubules during epithelial cell polarization [40] and its high level of expression has been reported in germ, Sertoli, and Leydig cells of the testis [39-40]. A fine mapping and positional cloning study indicated that a 13-kb deletion in *MAP7* was the causal mutation for microtubule stabilization in spermatogenesis in mice [41].

*EPB41L2* (also known as *protein 4.1G*) encodes a membrane skeletal protein [42], and a member of the protein 4.1 family originally identified in erythrocytes. *EPB41L2* is highly expressed in the testis of mice [42-43]. This protein may have a similar function as protein 4.1B, which is localized in the attaching region of spermatogonia and Sertoli cells and may have an important function in spermatogenesis in mice [44]. Another report showed that the absence of protein 4.1G could cause altered expression and localization of the cell adhesion molecule nectin-like 4 in testes, leading to male infertility in mice [45].

Several studies identified GJA1 (also known as connexin 43 or CX43) as the most abundantly expressed gap junction protein in the testicular cells of humans and several animals [46-49]. Previously, GJA1 was found to be essential in the control of male reproductive function, especially in spermatogenesis [50-58]. Various knockout mouse models have demonstrated the importance of CX43 in spermatogenesis [59]. In contrast with *CX43*, knockout mice for other *CX* genes are viable and no effects on spermatogenesis have been reported [60]. However, the molecular mechanism by which CX43 controls spermatogenesis has not been clearly identified. A recent study indicated that CX43 is involved in germ cell growth by controlling spermatogonia survival rather than proliferation [61]. Inhibition of adipocyte differentiation by Nur77, Nurr1, and Nor1 demonstrated that *GJA1* and *tolloid-like 1* (*Tll1*) are the Nur77-responsive genes. Retroviral expression of either *GJA1* or *Tll1* in 3T3-L1 preadipocytes also inhibited adipocyte differentiation, implicating these genes as potential mediators of the effect of Nur77 on adipogenesis [62].

GPRC6A is a member of family C of the G protein-coupled receptors (GPCR), which are closely related to the calcium-sensing receptor, CASR [63-66]. *GPRC6A* is broadly expressed in many tissues and organs, including lung, liver, spleen, heart, kidney, skeletal muscle, testis, brain, and bone [63-66]. In mice, *GPRC6A* was found to play an important role in the transduction of non-genomic effects of testosterone and other steroids [67]. The differential association of both insulin resistance and obesity with hypoandrogenism in men and hyperandrogenism in women suggests that androgens may exert sex-specific effects on adipose and other tissue. A recent study showed that androgens are essential for normal adipogenesis in males and can impair essential adipocyte functions in females. These findings strengthen the experimental basis for sex-specific effects of androgens in adipose tissue [68].

*TEX9* is highly expressed in the testis tissue of humans, but its function is not clear [69].

CYP19A1 is an enzyme belonging to the cytochrome P450 family. This enzyme is a specific heme-glycoprotein aromatase, which contains a steroid binding pocket [70-71]. Aromatase is widely distributed in the testis of mammals, including humans [71-72]. In adult men, transcripts of aromatase have been found in Leydig cells and Sertoli cells [71]. Additionally, CYP19A1 has been found in animal spermatozoa [73] and ejaculated spermatozoa from healthy men, and is associated with a stronger intensity of cytoplasmic droplets in sperm cells [71]. Aromatase transcripts were also detected in mouse bone, aorta, hypothalamus, adipose, gonads, and placenta, indicating that aromatase may be important in these tissues [74].

The *PDE8A* gene is a member of the *PDE8* family and is expressed in the ovary, testis, and other tissues including liver, kidney, and heart [75-79]. A previous study suggested that both *PDE8A* and *PDE8B* play essential roles in maintaining low cAMP levels, and then suppress the resting of steroidogenesis [80]. Another study showed that multiple *PDE* genes work in concert to regulate three of the important pathways leading to brown adipose tissue activation [81]. This finding may provide an improved conceptual basis for the development of therapies for obesity.

The *SH3GL3* gene, also known as *SH3P13*, is a BAR domain-containing protein and is expressed in the brain and testis tissue. *SH3GL3* is important in regulating clathrin-coated vesicle traffic, which is crucial for acrosome biogenesis during spermatogenesis [82].

From these known functions, we conclude that the remaining eight genes identified in the GWAS reported here may also have important effects on reproductive traits. Four of these genes, *GJA1*, *GPRC6A*, *CYP19A1,* and *PDE8A*, may not only affect the reproductive traits, but may also play a key role in the development of adipose tissue. For example, these genes may affect testis growth, which can result in an unusual level of hormone secretion and subsequent disruption of fat metabolism.
